# The Association Between Relationship Events and Experiences and Partner Evaluations: An Ideal Standards Perspective

**DOI:** 10.3389/fpsyg.2021.633267

**Published:** 2021-04-26

**Authors:** Susan Chesterman, Gery C. Karantzas, Emma M. Marshall

**Affiliations:** ^1^SoAR Laboratory, School of Psychology, Deakin University, Melbourne, VIC, Australia; ^2^SoAR Laboratory, School of Psychology, Deakin University, Geelong, VIC, Australia

**Keywords:** ideal standards, partner evaluation, romantic relationships, relationship events, discrepancy

## Abstract

Drawing on the Ideal Standards Model, the current study investigated whether the relationship events and experiences that occur on a given day in romantic relationships were associated with partner evaluations. Individuals in a current romantic relationship (*N* = 104) completed daily measures of positive and negative relationship events and experiences and partner evaluations for seven consecutive days. As hypothesized, findings demonstrated that on a given day negative relationship events and experiences were associated with evaluating partners as falling short of mate ideals, while positive relationship events and experiences were associated with evaluating partners as more closely meeting ideals. The findings demonstrate the importance of the relational context in evaluations of a partner against ideal standards.

## Introduction

Over the last two decades, many studies have investigated the effects of relationship events and experiences on people's evaluations of their romantic relationships (e.g., Neff and Karney, 2009). This research has found that negative daily relationship events (such as relationship conflicts) are associated with relationship dissatisfaction and dissolution (Campbell et al., 2005; Tolpin and Cohen, 2006). In contrast, positive daily relationship events (such as positive shared experiences or perceptions of partner responsiveness) are typically associated with relationship satisfaction and relationship maintenance (Gable et al., 2004; Girme et al., 2014). Although this research has provided important insights into how daily relationship events and experiences are associated with people's relationship evaluations, research has not investigated the role of these daily experiences in predicting partner evaluations. This is especially surprising given that partner evaluations have an important diagnostic function in predicting relationship satisfaction (Fletcher et al., 2000; Campbell et al., 2001; Karantzas et al., 2019), longevity (Fletcher et al., 1999), and the extent to which people try to change (i.e., regulate) a partner's relationship behavior (Overall et al., 2006).

To address this gap, the current paper reports on a daily diary study that draws on the Ideal Standards Model (ISM; Fletcher et al., 1999) of mate preferences to investigate how positive and negative relationship events and experiences (from this point on referred to as relationship events/experiences) are associated with romantic partner evaluations on a given day. The current study provides novel insights by being the first to investigate how positive and negative events affect partner evaluations on a daily basis.

Given that we frame the study of daily relationship events/experiences and partner evaluations within the ISM (Fletcher et al., 1999), we start by discussing the ISM and the conceptualization of partner evaluations within this framework. We then discuss how positive and negative daily relationship events/experiences are likely to be associated with partner evaluations.

### The Ideal Standards Model

The ISM (Fletcher et al., 1999; Fletcher and Simpson, 2000) is rooted within an evolutionary psychology perspective of mate preferences and mating strategies. According to this perspective, the qualities which are valued in a potential partner are those which contribute to reproductive fitness (i.e., good genes and parental investment; Gangestad and Simpson, 2000). Early empirical work into the ISM identified that individuals evaluate a mate's reproductive fitness across three broad partner ideals: warmth/trustworthiness (characteristics such as understanding, supportive), vitality/attractiveness (characteristics such as outgoing, charismatic) and status/resources (characteristics such as successful, financially secure; Fletcher et al., 1999). The extent to which a partner is evaluated as falling short on different ideal standards is termed *ideal-partner discrepancies*.

Ideal-partner discrepancies are therefore comprised of two components: (1) the importance that a person places on an ideal standard, and (2) the perception that a partner exhibits characteristics reflective of that ideal standard (Fletcher et al., 1999). The difference (or gap) between levels of ideal importance and partner perceptions reflects ideal-partner discrepancies. These discrepancies have important implications for how individuals evaluate their relationships. Smaller ideal-partner discrepancies have been found to be associated with higher levels of relationship satisfaction and a lower likelihood of relationship dissolution (Fletcher et al., 2000; Campbell et al., 2001; Buyukcan-Tetik et al., 2017). In contrast, larger ideal-partner discrepancies are associated with lower relationship satisfaction and greater attempts at regulating a partner's behavior as a way to reduce the size of the discrepancy (Overall et al., 2006, 2009). That is, when a partner is evaluated as falling short of ideals, individuals try to change their partner's behavior in a way that the partner's qualities more closely match an individual's ideal standards (Fletcher et al., 1999; Campbell and Fletcher, 2015). In this way, areas in which a partner is evaluated as falling below what is desired can be addressed rather than having issues escalate and dissatisfaction with the partner increase.

### Ideal-Partner Discrepancies and Relationship Events/Experiences

If ideal-partner discrepancies indeed function to provide an individual with important diagnostic information regarding how well a partner meets one's relational needs, then ideal-partner discrepancies should covary as a function of the events and experiences that people encounter as part of their relationships on a daily basis. Negative relationship events/experiences should be associated with people negatively evaluating their partners, and as such, deem partners as falling short of ideals. For example, on days when relationship conflict ensues or support exchanges lack sensitivity, then partners should be evaluated as falling short on characteristics associated with warmth/trustworthiness such as understanding and kindness. Likewise, relationship events/experiences that reflect dissatisfying sexual interactions with one's partner or difficulties with managing finances may heighten evaluations that a partner falls short on characteristics associated with the ideal dimension of vitality/attractiveness or status/resources.

On the other hand, positive relationship events/experiences should prime individuals to reflect on the extent that partners ably meet a person's needs and wants, and thus evaluations of partners should be more positive—an indication that partners exhibit characteristics that more closely align with ideal standards. For instance, a highly responsive partner, or one who engages in constructive conflict behaviors, should be evaluated as meeting or exceeding a person's expectations regarding warmth/trustworthiness. Similarly, highly satisfying sexual experiences or committing to shared financial goals should enhance evaluations of a partner as meeting one's ideals around vitality/attractiveness or status/resources.

However, many relationship events and experiences are unlikely to be exclusively associated with the evaluation of partners along a single ideal-dimension. For instance, if a couple has a dinner date at a fine-dining restaurant in formalwear or smart attire, then each individual may evaluate their partner positively in terms of meeting their ideals of vitality/attractiveness as well as status/resources.

The relevance of the relationship event/experience to the individual should also determine the degree to which a particular event will be associated with larger or smaller ideal-partner discrepancies. In their theory of stress appraisal, Lazarus and colleagues noted that regardless of whether the events are major life events or daily hassles, the appraisal of an event's significance should provide information regarding the degree to which the event is relevant to the individual (Lazarus and Folkman, 1984; Lazarus, 2006). For instance, a partner not providing help with domestic duties may be appraised as a more or less significant relationship event/experience, depending on how relevant a partner helping with domestic duties is to the individual.

Despite these predictions, research to date has not investigated the role of relationship events/experiences in predicting daily partner evaluations. However, insights as to the role of relationship events/experiences can be drawn from diary studies into the associations between such events and relationship evaluations. In a series of four 7-day diary studies, Neff and Karney (2009) examined the co-variation in daily relationship experiences such as the couples sex life, time spent together, their conversations and the way disagreements were resolved. The results revealed that on average, and at the level of the individual, these daily relationship events/experiences were significantly positively associated with global relationship evaluations (Neff and Karney, 2009). Similarly, in a diary study over 10 days, negative relationship events (such as having an unresolved argument) were found to be associated with decreased levels of daily relationship satisfaction for dating partners (Tolpin and Cohen, 2006). This study also found positive relationship events such as outings and socializing with one's partner were associated with increased levels of relationship satisfaction. Furthermore, in a 14-day diary study of same-sex romantic couples, Ogolsky and Gray (2016) found that experiencing higher perceived levels of daily conflict was associated with lower levels of the evaluation of relationship maintenance.

Extending on this research to focus on partner evaluations can provide insights regarding how relationship events/experiences are associated with people's diagnostic assessment of partners. This can have subsequent implications for how people engender change to redress ideal-partner discrepancies or enact maintenance behaviors when partners meet mate standards.

### De-composing the Association Between Ideal-Partner Discrepancies and Relationship Events/Experiences

As already noted, ideal-partner discrepancies can be conceptualized as the difference between the importance placed on an ideal standard and the perception that a partner exhibits this ideal. Thus, ideal-partner discrepancies entail two constituent parts. An important question that emerges is which component is more (or less) associated with positive and negative relationship events/experiences. One the one hand, relationship events/experiences may be associated with larger or smaller ideal-partner discrepancies because these events are associated with the calibration of the ideal itself. That is, relationship events/experiences may be tied to the importance people place on ideal standards. Indeed, Fletcher and colleagues (Fletcher et al., 1999; Fletcher and Simpson, 2000) note that ideals are, in part, derived through romantic relationship experiences. However, Fletcher and colleagues also note that ideals have an evolutionary underpinning and are knowledge structures derived through more than just relationship experiences (Fletcher et al., 1999; Fletcher and Simpson, 2000). Rather, ideal standards reflect schemas that integrate knowledge about the self (i.e., personal characteristics that overlap with ideals [e.g., understanding, funny, good looking]), as well as knowledge that relates to relationships more generally (i.e., social learning through observing the relationships of close others as well as socio-cultural views of relationships; Fletcher et al., 1999). Thus, a case can be made that relationship events/experiences may demonstrate small (if any) associations with ideals, given the assumed multifaceted nature of ideal standards.

On the other hand, relationship events/experiences may be associated with partner perceptions. As such, relationship events/experiences may correspond to the extent that individuals notice their partner exhibits particular ideal characteristics. Alternatively, it is plausible that relationship events/experiences are associated with both constituent parts, and thus, ideal-partner discrepancies reflect the raising or lowering of ideal standards and perceiving partners as exhibiting more or less of a given ideal. Investigating the extent to which relationship events/experiences are related to ideal importance and/or partner perceptions has important implications for understanding the processes that underpin partner evaluation and the role of the relational context in these evaluative processes.

Despite the implications of addressing this gap, there is a dearth of research regarding the associations between relationship events/experiences and the constituent parts of ideal-partner discrepancies. In the only study on this topic, Bredow and Hames (2018) found that changes in ideal standards (over a 3 year period) were a function of positive and negative relationship events, across the three ideals. Specifically, it was found that individuals who experienced more positive events (e.g., becoming engaged) displayed greater increases in their standards regarding partner vitality/attractiveness and warmth/trustworthiness. Further, individuals who experienced more negative events (e.g., partner infidelity) had less increase in the status/resources ideal over time. However, a limitation of this study noted by the authors was that the assessment of events and mate standards were too infrequent to capture the many and varied relationship experiences that occur on a weekly or daily basis that may feed into the calibration of mate standards. Moreover, the study did not investigate the extent to which relationship events moderated partner perceptions or ideal-partner discrepancies.

### Overview of the Current Study

To address the dearth of research into the association between relationship events/experiences and ideal-partner discrepancies, the current study had two aims. The first aim were to determine whether positive and negative events/experiences are associated with ideal-partner discrepancies on a given day. Specifically we hypothesized that, on a given day: (1) a more significant positive relationship event/experience would be associated with smaller ideal-partner discrepancies across all ideal dimensions and (2) a more significant negative relationship event/experience would be associated with larger ideal-partner discrepancies across all ideal dimensions. The second aim was to determine whether associations between events and ideal-partner discrepancies on a given day were a function of the associations between these events and the ideal standard itself and/or the perceptions of the partner along the ideal dimensions. Given that, on balance, it is equally plausible for relationship events/experiences to be: (a) associated with ideal standards or (b) have no association with ideals, we made no specific predictions regarding the association between the ideal standard itself and relationship events/experiences. However, in terms of partner perceptions, we hypothesized that (3) a more significant positive relationship event/experience would be positively associated with partner perceptions on a given day, and (4) a more significant negative relationship event/experience would be negatively associated with partner perceptions on a given day.

## Method

### Participants

Participants were 104 individuals (76.7 % women, 23.3% men, *M* = 27.28 years old, *SD* = 8.34 years [ranging from 18 to 60 years]) in a current, romantic relationship for at least 6 months^1^. The sample was comprised of 30.8% married or engaged, 67.2% cohabitating or steady dating and 1% casually dating participants with 91% identifying their relationship as heterosexual. The average relationship duration was 4.21 years (*SD* = 5.57) and 87.2% of participants were of Anglo-Saxon background.

### Procedure and Measures

This study was approved by Deakin University's ethics committee. Interested individuals who read social media (Facebook and Reddit) advertisements about the study followed a URL link that directed them to a website with study details. On registering their interest through the website, participants received a return email with a Plain Language Statement and calendar detailing the dates that they would receive the survey. Participants were advised that their consent to participate in this study was implied by beginning the first online survey. A link to the daily survey was emailed and texted to each participant at (approximately) 6 P.M. each day over the 7-day period with instructions to complete the survey that night, however, the survey was left open until 12 p.m. of the following day for late entries. All measures were completed on each day.

#### Ideal-Partner Discrepancies (Including Ideal Partner Standards and Partner Perceptions)

The importance of partner ideal standards and the extent to which partners are perceived to exhibit these ideals was assessed using an adapted version of the Ideal Standards Scale-Short Form (ISS-SF; Fletcher et al., 1999). The adapted measure consisted of six partner characteristics selected on their face validity. Two items assessed each of the ideal subscales of warmth/trustworthiness (characteristics of understanding and supportiveness), vitality/attractiveness (outgoing and charismatic) and status/resources (good job and financially secure^2^). All six items were rated by participants twice. In the first instance participants rate the importance of each ideal partner characteristic on a scale ranging from 1 (very unimportant) to 7 (very important). In the second instance, participants rate the extent to which a current romantic partner is perceived to exhibit each of the six ideal characteristics on a 7-point scale ranging from 1 (not at all like my partner) through to 7 (very much like my partner). To adapt this measure to a daily-diary methodology, instructions from the original ISS-SF were slightly re-worded to capture ideal partner importance and partner perception ratings for the day they were reported. Ideal partner scales demonstrated high internal reliability (see Table 1). Ideal-partner discrepancies were derived for warmth/trustworthiness, vitality/attractiveness and status/resources by regressing mean levels of ideal importance onto mean levels of partner perceptions (see Data Analysis section for details).

**Table 1:** Descriptive statistics for each person's average score over 7 days.

**Variable**	**1**	**2**	**3**	**4**	**5**	**6**	**7**	**8**	**9**	**10**	**11**
Import W/T	1.00										
Import V/A	0.14^**^	1.00									
Import S/R	0.22^**^	0.29^**^	1.00								
Percept W/T	0.23^**^	0.10^*^	0.18^**^	1.00							
Percept V/A	0.19^**^	0.45^**^	0.12^**^	0.38^**^	1.00						
Percept S/R	0.00	0.08^*^	0.04	0.22^**^	0.22^**^	1.00					
Discrep W/T	0.00	0.07	0.13^**^	0.96^**^	0.33^**^	0.23^**^	1.00				
Discrep V/A	0.14^**^	0.00	−0.01	0.37^**^	0.88^**^	0.20^**^	0.33^**^	1.00			
Discrep S/R	−0.01	0.08^*^	0.00	0.21^**^	0.21^**^	0.99^**^	0.22^**^	0.20^**^	1.00		
PosEvent	−0.08	0.13^*^	0.00	0.05	0.11^*^	−0.06	0.07	0.04	−0.07	1.00	
NegEvent	0.02	0.13	0.04	−0.17	0.02	−0.08	−0.19	−0.02	−0.09	0.49^**^	1.00
Mean	6.57	4.56	4.90	6.08	5.07	5.38	0.00	0.00	0.00	4.38	4.10
Standard deviation	0.80	1.45	1.46	1.29	1.49	1.44	0.99	0.99	0.99	1.72	2.07
Reliability			0.97	0.85	0.96	0.98	0.89	0.97			

*PosEvent, positive event; NegEvent, negative event; Import W/T, Ideal importance warmth/trustworthiness; Import V/A, Ideal importance vitality/attractiveness; Import S/R, Ideal importance status/resources; Percept W/T, Partner perceptions warmth/trustworthiness; Percept V/A, Partner perceptions vitality/attractiveness; Percept S/R, Partner perceptions status/resources; Discrep W/T, Ideal-partner discrepancies warmth/trustworthiness; Discrep V/A, Ideal-partner discrepancies vitality/attractiveness; Discrep S/R, Ideal-partner discrepancies status/resources; Reliability, estimate of within-person reliability (Cranford et al., 2006) R_C_*.

* *p < 0.05*,

** *p < 0.01*.

#### Daily Events

The two daily event categories: (1) positive events relating to the partner and/or relationship and (2) negative events relating to the partner and/or relationship were assessed via a series of questions. For example, participants were asked to respond to the statement “Today I had a positive experience that was related to my partner or was related to my relationship.” If participants answered “yes,” they were then asked to briefly describe the event. These responses were not used in the data analysis. Finally, participants were asked to rate the significance of the event (i.e., “this event was significant”) on a scale of 1 (strongly disagree) to 7 (strongly agree). The significance rating of the event was used as part of analyses in the current study. From the significance rating we created two variables to allow us to examine within person effects while controlling for between-person effects (see Data Analysis section).

The daily surveys were identical to each other with the exception of the first survey, which also included demographics. As an incentive to participate and to minimize attrition, participants were given a set of individually tailored graphs of how their ratings on certain variables tracked over time at the completion of the study.

### Data Analysis

Ideal-partner discrepancies were derived for warmth/trustworthiness, vitality/attractiveness and status/resources as residual scores by regressing partner perception scores onto ideal importance scores for each of the three ideal domains^3^. This method of deriving ideal-partner discrepancies has been used in past research on the ISM (e.g., Overall et al., 2006; Karantzas et al., 2019). The significance ratings of the positive and negative relationship event/experiences were entered as predictors in the analyses. To test whether daily events were associated with ideal-partner discrepancies at a within-person level, we ran a series of multilevel models (Bolger and Laurenceau, 2013) using SPSS mixed procedure (Version 23; IBM Corp, 2015). Repeated daily assessments (7 time points) of positive and negative daily relationship events/experiences and ideal-partner discrepancies (Level 1) were nested within participants (Level 2). First, we ran three models for ideal-partner discrepancies; one for each of the three ideal dimensions. To examine within-person associations between ideal-partner discrepancies and events, event variables were person centered. In addition, to control for between-person associations, we reintroduced the mean by adding between-person mean event variables to the model (Curran and Bauer, 2011; Bolger and Laurenceau, 2013). The between-person event variables were computed by averaging the responses for all cases for that person, thereby creating a group-level variable with one value for each person. All event variables were entered as fixed effects and time entered as a random effect to account for the repeated measurement and non-independence of the data. Maximum likelihood estimation was applied to the data (Bolger and Laurenceau, 2013). An unstructured covariance structure was applied to residuals for the random effect of time as we could not achieve convergence with other covariance structures^4^. To determine the source of the significant associations between events and ideal-partner discrepancies, we then ran the significant models twice, this time with ideal importance or partner perceptions as dependent variables.

We also conducted a series of Supplementary Analyses to test for any lagged effects regarding relationship events/experiences and ideal-partner discrepancies. The lagged analyses enable us to determine whether events reported on the previous day were associated with ideal-partner discrepancies on the following day. As part of these analyses, we re-ran the original three models using ideal-partner discrepancies as a dependent variable but included positive and negative relationship events/experiences as lagged variables (i.e., T-1).

An apriori power analysis was conducted using Power analysis IN Two-level designs (PiNT, 2.12, Snijders et al., 2007) indicating a sample size of *N* = 100 to detect small effects in a two-level model would be adequately powered (0.83).

## Results

### Descriptive Statistics

Means, standard deviations, reliability statistics and correlations for all variables at are presented in Table 1. The mean levels of ideal importance were moderate for vitality/attractiveness and status/resources and high for warmth/trustworthiness. Levels of partner perceptions were moderate to high for each of the ideal dimensions. Participants' reported moderate levels of significance for both positive and negative daily events. Correlations between the variables showed that although ideal importance and ideal-partner discrepancies were not significantly associated, partner perceptions and ideal-partner discrepancies had low to moderate associations. Associations between partner perceptions and ideal-partner discrepancies on the same ideal dimension were high.

### Associations Between Daily Events and Ideal-Partner Discrepancies

We first examined whether daily relationship events/experiences were associated with ideal-partner discrepancies over the 7 day period (see Table 2). Findings revealed that on days when individuals experienced a more significant positive relationship event/experience, individuals evidenced smaller ideal-partner discrepancies across all three ideal dimensions. In terms of negative events, results showed that on days when individuals experienced a more significant negative relationship event/experience, individuals evidenced larger ideal-partner discrepancies across all three ideal dimensions.

**Table 2:** Parameter estimates.

		**Estimate**	**SE**	***p***	**CI LL**	**CI UL**	**Beta**	***r***
**Warmth/trust**
I-P discrepancy	Pos event	0.079	0.013	<0.001	0.053	0.105	0.007928	0.058
	Pos event between	0.085	0.041	0.039	0.005	0.165	0.026339	0.076
	Neg event	−0.141	0.016	<0.001	−0.173	−0.110	−0.01742	−0.067
	Neg event between	−0.288	0.073	<0.001	−0.432	−0.144	−0.1601	−0.021
Ideal importance	Pos event	−0.009	0.010	0.353	−0.029	0.011	−0.0008	−0.051
	Pos event between	−0.016	0.037	0.667	−0.089	0.057	−0.00492	−0.055
	Neg event	0.003	0.012	0.782	−0.021	0.028	0.000351	0.050
	Neg event between	0.142	0.065	0.032	0.013	0.271	0.0778	0.128
Partner perceptions	Pos event	0.104	0.017	<0.001	0.072	0.137	0.009747	0.060
	Pos event between	0.094	0.055	0.093	−0.016	0.204	0.029142	0.079
	Neg event	−0.178	0.020	<0.001	−0.218	−0.138	−0.02035	−0.070
	Neg event between	−0.323	0.099	0.002	−0.520	−0.127	−0.17949	−0.229
**Vitality/attract**
I-P discrepancy	Pos event	0.044	0.012	<0.001	0.020	0.068	0.003371	0.053
	Pos event between	−0.003	0.050	0.953	−0.102	0.096	−0.00092	−0.051
	Neg event	−0.049	0.015	0.001	−0.078	−0.019	−0.00456	−0.055
	Neg event between	0.035	0.089	0.692	−0.141	0.211	0.019485	0.069
Ideal importance	Pos event	0.001	0.011	0.918	−0.021	0.024	0.00004	0.050
	Pos event between	−0.066	0.082	0.426	−0.229	0.098	−0.02035	−0.070
	Neg event	−0.007	0.014	0.621	−0.034	0.021	−0.00036	−0.050
	Neg event between	0.029	0.147	0.846	−0.262	0.320	0.015791	0.066
Partner perceptions	Pos event	0.064	0.017	<0.001	0.031	0.096	0.004193	0.054
	Pos event between	−0.015	0.078	0.845	−0.170	0.139	−0.00473	−0.055
	Neg event	−0.080	0.020	<0.001	−0.120	−0.040	−0.00649	−0.056
	Neg event between	0.058	0.139	0.676	−0.217	0.334	0.032186	0.082
**Status/resources**
I-P discrepancy	Pos event	0.021	0.008	0.005	0.007	0.036	0.000885	0.051
	Pos event between	−0.001	0.056	0.984	−0.112	0.110	−0.00035	−0.050
	Neg event	−0.019	0.009	0.044	−0.037	−0.001	−0.00089	−0.051
	Neg event between	−0.102	0.100	0.311	−0.300	0.096	−0.05608	0.106
Ideal importance	Pos event	−0.006	0.012	0.638	−0.029	0.018	−0.00025	−0.050
	Pos event between	−0.011	0.082	0.894	−0.174	0.152	−0.00339	−0.053
	Neg event	−0.026	0.014	0.074	−0.054	0.003	−0.00139	−0.051
	Neg event between	0.017	0.146	0.908	−0.273	0.306	0.00932	0.059
Partner perceptions	Pos event	0.028	0.011	0.010	0.006	0.049	0.001126	0.051
	Pos event between	0.008	0.081	0.919	−0.153	0.170	0.002573	0.053
	Neg event	−0.028	0.013	0.034	−0.054	−0.002	−0.00141	−0.051
	Neg event between	−0.147	0.145	0.313	−0.434	0.140	−0.08082	−0.131

*The columns represent the estimates and standard errors for fixed effects of the predictor being examined. Warmth/trust, ideal dimension warmth/trustworthiness; Vitality/attract, ideal dimension vitality/attractiveness; Status/resources, ideal dimension status/resources; I-P discrepancy, Ideal-Partner discrepancy; Pos event, positive event; Neg event, negative event; Pos event between, between-person positive event; Neg event between, between-person negative event; CI LL 95% confidence interval lower limit; CI UL 95% confidence interval upper limit; r, effect size*.

### De-composing the Association Between Ideal-Partner Discrepancies and Relationship Events/Experiences

Next we explored whether the source of the significant associations between individuals' positive and negative relationship events/experiences and ideal-partner discrepancies was related to ideal importance and/or partner perceptions. We ran the same models used to assess the associations between events/experiences and ideal-partner discrepancies over 7 days, this time using ideal importance and partner perceptions as dependent variables across three ideal dimensions, respectively.

The associations between positive and negative relationship events/experiences and ideal importance ratings across the three ideal dimensions were non-significant. In terms of partner perceptions, on days when individuals experienced a more significant positive relationship event/experience, they evidenced higher perceptions of their partners across all three ideal dimensions. On days when individuals experienced a more significant negative relationship event/experience, they evidenced lower perceptions of their partners across all three ideal dimensions.

### Lagged Analyses

Supplementary Analyses were conducted to test for lagged effects regarding relationship events/experiences. Specifically, we tested for whether positive or negative relationship events/experiences on the previous day were associated with ideal-partner discrepancies on the following day, using lagged (T-1) event variables. These analyses demonstrated no significant associations between events occurring on 1 day and ideal-partner discrepancies on the following day (see Supplementary Material 1).

## Discussion

The current study is the first to demonstrate that daily relationship events/experiences are associated with the extent that a romantic partner is evaluated as falling short or meeting an individual's ideal standards. As hypothesized, positive relationship events/experiences were associated with smaller ideal-partner discrepancies across all ideal dimensions on a given day. Also in line with predictions, negative relationship events/experiences were associated with larger ideal-partner discrepancies across all ideal dimensions on a given day. Supplementary Analyses testing for lagged effects revealed that the positive and negative relationship events/experiences reported on a given day were not associated with ideal-partner discrepancies on the following day.

The findings extend understanding as to how relationship events/experiences are associated with the components that comprise ideal-partner discrepancies; we found that events were associated with partner perceptions rather than the ideal standards themselves. Thus, the association between positive and negative relationship events/experiences and ideal-partner discrepancies is such that individuals maintain their level of ideal importance irrespective of the events experienced on a given day. However, daily events figure into people's perceptions of their partners, which determines the extent to which partners are evaluated as falling short of ideals.

The findings suggest that the relationship events/experiences that occur for individuals on a given day are internalized and reflected in the evaluations of relationship partners. In particular, positive relationship events/experiences are associated with smaller ideal-partner discrepancies whereas negative relationship events/experiences are associated with larger ideal partner-discrepancies. The findings provide support for the notion that people's perceptions of their partner tracks in relation to new information (Fletcher and Kerr, 2010; Fletcher, 2015). Although people may also maintain a positive or negative bias when making judgments of their partners and/or relationships, this has been found to operate quite separately from their ability to accurately track changes (Fletcher and Kerr, 2013). From an evolutionary standpoint, partner perceptions should demonstrate some correspondence with ideal partner dimensions of warmth/trustworthiness, vitality/attractiveness, and status/resources to ensure that individuals maintain relationships with partners who reflect optimal mates regarding reproductive fitness; otherwise, these associated characteristics could not have evolved by way of natural and sexual selection (Fletcher, 2015).

What are the implications regarding the correspondence between relationship events/experiences and partner evaluations on a given day? This correspondence may help individuals to manage risk and rewards in romantic relationships (Murray et al., 2006, 2008). According to Murray and colleagues, relationships inherently encompass regulating risks and rewards regarding the probability of rejection and the likelihood of love and acceptance. Thus, relationship circumstances can motivate an individual to either prioritize self-protection (i.e., avoid relational threats and punishments) or connectedness (i.e., approach relationship rewards) as a way to either mitigate rejection or to optimize love, safety and acceptance in their romantic relationships. Thus, negative relationship events/experiences may on the one hand signal to an individual that their sense of safety may be in jeopardy and that they may be at greater risk of some negative consequence. On the other hand, positive events may signal opportunities for love, security and human connection.

Thus, relationship events/experiences provide important contextual information about one's relationship that acts as an input into people's judgements regarding the likelihood that a partner may enact relational punishments or rewards. That is, events that correspond with a mate being evaluated as falling short on ideal standards may convey that the partner is more of a risk in either not meeting a person's socio-emotional needs and/or leveling punishments such as in the form of rejection and betrayal. For instance, if the experience of hurtful comments from a partner corresponds with the evaluation of them falling short of “kindness” or “supportiveness” ideals, then this may convey the risk of immediate or future rejection by the partner. In contrast, events that correspond with partners being evaluated as meeting ideal standards are unlikely to be evaluated as a risk, but rather, as a mate who is well-suited to meet one's socio-emotional needs. For instance, if the experience of a partner showing empathy corresponds with the evaluation of them as meeting ideal standards in “understanding” or “supportiveness,” then this may convey a sense of opportunity for rewards such as closeness and emotional connection in the relationship.

This correspondence (i.e., association) between relationship events/experiences and partner evaluations may also have important implications regarding broader relationship goals and transitions. For instance, during periods such as when a couple prioritizes to start a family, relationship events that correspond with positive evaluations of partners along dimensions such as vitality/attractiveness may increase sexual behavior thereby heightening the likelihood of successful conception. Likewise, events that correspond to the positive evaluations of partners in relation to warmth/trustworthiness and status/resources may heighten motivations to commence a family with a partner, as the qualities they exhibit reflect highly desirable characteristics regarding parental commitment/investment. Importantly, the fact that positive relationship events/experiences correspond with positive evaluations of relationship partners highlights the function of these evaluative judgments in ensuring that relationship rewards, pleasures and needs are acknowledged. This acknowledgment of relationship positivity—in terms of events and partner evaluations—is important given that research highlights the ratio of positive relationship experiences to negative experiences is more important than the absence of negative experiences *per se* (Gottman and Levenson, 1992; Rusbult et al., 2001).

Another important finding from the current study that has important implications for understanding partner evaluations relates to identifying the component of the ideal partner-discrepancy which is most associated with relationship events/experiences. Our results revealed that relationship events/experiences were significantly associated with partner perceptions rather than the ideal standards themselves. Thus, individuals appear to perceive their partners as exhibiting (more or less) ideal mate characteristics as a function of the positivity or negativity of the relational events/experiences of a given day. But why wouldn't these events/experiences be associated with the importance people place on the ideal standards?

Firstly, ideal standards are considered mate criteria (Campbell and Fletcher, 2015). Typically, criteria function as benchmarks that aide in making effective judgments (Thibaut and Kelley, 1959). In the case of partner ideals, these standards afford individuals to make judgements as to whether a partner is indeed a suitable mate. Thus, if the ideal standards themselves were found to increase and decrease on a daily basis as a function of the events experienced, people would inherently have difficulties in making astute evaluations of partners over the course of a relationship. That is, the “standard” provides information and a level of certainty about what is desired in a mate and the relative evaluations that people can make about one's partner (Fletcher and Simpson, 2000). Furthermore, theory regarding the development of people's ideal standards suggests that ideals reflect knowledge structures that comprise self-knowledge as well as knowledge about relationships accumulated across time and society more generally (e.g., Fletcher et al., 1999; Fletcher and Simpson, 2000; Campbell and Fletcher, 2015). Thus, ideals standards reflect broad knowledge structures that may be quite resistant to increases and decreases on a daily basis, despite the positivity or negativity of relationship events on a given day. However, ideals may demonstrate some shifts over extended periods of time, especially when individuals experience major or highly significant relationship events or transitions that require them to re-consider their mate standards. In support of this, Bredow and Hames (2018) found that major life events such as becoming engaged or experiencing a partner's betrayal (in the form of infidelity) moderated change in ideal standards over time. Thus, ideal standards may be associated with relationship events, however, these associations may only become evident over longer timespans, when individuals have actively revised their knowledge structures around mate preferences because of major events that have challenged existing standards.

### Limitations and Future Directions

Although the current study has added to our understanding of ideal-partner discrepancies, the results should be viewed in light of the following limitations. Firstly, the context within which a romantic relationship takes place is complex and the current study was limited in the way it measured daily events. Assessing the “significance” of an event is only one facet of whether an event may impact ideal-partner discrepancies. Although the significance of an event may be high, it is an individual's initial cognitive appraisal of how distressing or rewarding the event is that may also be associated with their subsequent evaluation and reaction to it (Folkman et al., 1986). Future studies should endeavor to capture a measure of the level of distress experienced as a result of negative events and a measure of reward/positive affect for positive events. We also note the measurement of the two components of ideal-partner discrepancies uses slightly different wording in the scales. Ideal standards are assessed by participants rating the importance of characteristics in their ideal partner and partner perceptions by rating the extent to which these characteristics are exhibited/demonstrated by their current partner. Although some suggest that assessing the importance and perception may not reflect equivalent scales upon which to derive ideal-partner discrepancies (Gerlach et al., 2017) the use of these scales is consistent with the majority of research on ideal standards, as well as the mate preferences literature more generally (Buss, 1989; Fletcher et al., 1999; Eastwick and Neff, 2012).

Secondly, our sample was predominantly (77%) female which did not allow us to explore possible gendered effects. This is despite research findings that females hold ideal standards in the warmth/trustworthiness and status/resources dimensions as somewhat more important compared to males (Campbell et al., 2001). Future studies with a more representative sample would be a useful next step in this line of research to assess the extent to which gender may moderate associations between daily events and ideal-partner discrepancies.

Third, to guard against missing data bias and likelihood of attrition which can be experienced in daily diary studies (Gable et al., 2000; Scollon et al., 2003) we left the survey open until 12 p.m. of the following day. Only eight per cent of surveys were submitted on the following day. It is possible that the people responding on the following day may have differed in their recall of the previous day's events. However, any differences are unlikely to have affected the findings given the small percentage of surveys involved. This limitation does however highlight the need to balance the maintenance of a high level of participation with the amount of time available to complete surveys in daily diary studies.

Finally, it is difficult to disentangle the partner and relational aspects of the events, largely due to the interdependent nature of relationships (Rusbult and Van Lange, 2003). Oftentimes particular events, even when involving the partner, also have a relational focus. We asked about events and experiences related to the partner and/or relationship to capture a broad-based sense of the events which may impact romantic relationships. However, there is evidence to suggest that people hold cognitive representations of the partner and relationship separately (Brunson, 2014). It may be that events specific to the partner have a greater correspondence with partner evaluations and that relational events correspond with relationship evaluations. Thus, future research could attempt to include more fine-grained measures that can effectively uncouple assessments of partner and relationship events.

## Conclusion

This study is the first to empirically demonstrate that the daily events and experiences in romantic relationships are indeed associated with the degree to which a partner is evaluated as falling short of ideal standards on a given day. Not only do negative relationship events/experiences appear to be deleterious to partner evaluations, we also found that positive relationship events/experiences enhance partner evaluations. The associations between relationship events/experiences and partner evaluations demonstrates the importance of the daily relational context in the judgments of romantic partners.

## Data Availability Statement

Data can be accessed upon request in line with the conditions around Deakin Research Human Ethics approval. Requests should be directed to: smches@deakin.edu.au.

## Ethics Statement

The studies involving human participants were reviewed and approved by Deakin University Human Research Ethics Committee. The participants provided their written informed consent to participate in this study.

## Author Contributions

SC co-designed the study, collected the data, conducted the analyses, and wrote the first draft of the paper. GK co-designed the study, guided the data analyses, and edited the paper. EM guided the data analyses and edited the paper. All authors contributed to the article and approved the submitted version.

## Conflict of Interest

The authors declare that the research was conducted in the absence of any commercial or financial relationships that could be construed as a potential conflict of interest.

## Footnotes

^1^Initial recruitment resulted in a sample of 326, however 222 of these cases were excluded because they had 40% or greater of missing data.

^2^We added the words “or potential to achieve” to the status/resources characteristics to account for the likelihood that our sample age would be skewed toward younger participants (i.e., financially secure [or potential to achieve]; Overall et al., 2006).

^3^Calculating ideal-partner discrepancies in this way has an advantage over creating a difference score as it does not confound the partner perception and ideal standard components by controlling for ideal standard ratings.

^4^We initially ran these models with person-centered event variables also modeled as random effects, time as a repeated statement and an autoregressive covariance structure to the repeated statement [as recommended by Bolger and Laurenceau (2013)]. However, we could not achieve convergence and further investigation revealed extremely small covariances of the random slopes (Est = < 0.0001). Therefore, the events variables were excluded as random effects, resulting in the final approach to covariance/variance modeling that is described in Data Analysis. See Supplementary Material 1 for an example of the syntax for the random effects and current models.
