# Supplementary material for: The Association Between Relationship Events and Experiences and Partner Evaluations: An Ideal Standards Perspective
Source: Front Psychol. 2021 Apr 26;12:633267. doi: 10.3389/fpsyg.2021.633267 (PMC8107208; doi:10.3389/fpsyg.2021.633267)
Supplement: Supplementary file 1 [file Data_Sheet_1.docx]

**The Association Between Relationship Events and Experiences and Partner Evaluations: An Ideal Standards Perspective**

**Supplementary material**

**Syntax Random Effects example (would not converge)**

MIXED ISM_IMP_WT WITH time_c p_event_pc pos_event_mean n_event_pc neg_event_mean

/FIXED = p_event_pc pos_event_mean n_event_pc neg_event_mean

/PRINT = SOLUTION TESTCOV COVB G

/RANDOM INTERCEPT time_c p_event_pc n_event_pc | SUBJECT(id) COVTYPE(UN)

/REPEATED time_c SUBJECT(id) COVTYPE(AR1)

/CRITERIA=CIN(95) MXITER(1000) MXSTEP(50) SCORING(1) SINGULAR(0.0000000001) HCONVERGE(0,

ABSOLUTE) LCONVERGE(0, ABSOLUTE) PCONVERGE(0.000001, ABSOLUTE)

/METHOD = ML.

**Syntax Current model example**

MIXED ISM_IMP_WT WITH time_c p_event_pc pos_event_mean n_event_pc neg_event_mean

/FIXED = p_event_pc pos_event_mean n_event_pc neg_event_mean

/PRINT = SOLUTION TESTCOV COVB G

/RANDOM INTERCEPT time_c | SUBJECT(id) COVTYPE(UN)

/CRITERIA=CIN(95) MXITER(1000) MXSTEP(50) SCORING(1) SINGULAR(0.0000000001) HCONVERGE(0,

ABSOLUTE) LCONVERGE(0, ABSOLUTE) PCONVERGE(0.000001, ABSOLUTE)

/METHOD = ML.

Table 1

Lagged Analyses (T-1) Parameter Estimates

|  |  | Estimate | SE | p | CI LL | CI UL |
| --- | --- | --- | --- | --- | --- | --- |
| I-P discrep W/T | Pos event | -0.005 | 0.016 | 0.766 | -0.036 | 0.027 |
|  | Neg event | 0.003 | 0.020 | 0.870 | -0.035 | 0.042 |
| I-P discrep V/A | Pos event | -0.020 | 0.015 | 0.178 | -0.049 | 0.009 |
|  | Neg event | -0.003 | 0.018 | 0.853 | -0.039 | 0.032 |
| I-P discrep S/R | Pos event | -0.014 | 0.009 | 0.124 | -0.031 | 0.004 |
|  | Neg event | -0.006 | 0.011 | 0.558 | -0.028 | 0.015 |

*Note*. I-P discrep W/T = Ideal-Partner discrepancy warmth/trustworthiness, I-P discrep V/A = Ideal-Partner discrepancy vitality/attractiveness, I-P discrep S/R = Ideal-Partner discrepancy status/resources, Pos event = positive relationship event/experience and Neg event = negative relationship event/experience.

Table 2

Measures used in daily diary

____________________________________________________________________________________________________

**Positive** **relationship event/experience**

_____________________________________________________________________________________________________

Today I had a positive experience that was related to my partner, or was related to my relationship.

- Yes
- No

This experience was significant. 1 = strongly disagree to 7 = strongly agree

­­­­­­­­­­­­­­­­­­­­___________________________________________________________________________

**Negative relationship event/experience**

___________________________________________________________________________

Today I had a negative experience that was related to my partner, or was related to my relationship.

- Yes
- No

This experience was significant. 1 = strongly disagree to 7 = strongly agree

___________________________________________________________________________

**Ideal standard importance**

___________________________________________________________________________

Thinking about today, rate the following in terms of their importance in describing your IDEAL PARTNER in a romantic relationship: 1 = very unimportant to 7 = very important

- Understanding
- Good job (or potential to achieve)
- Charismatic
- Supportive
- Financially secure (or potential to achieve)
- Outgoing

___________________________________________________________________________

**Perceptions of the current partner**

___________________________________________________________________________

Thinking about today, rate the following in terms of their importance in describing your IDEAL PARTNER in a romantic relationship:  1 = not at all like my partner to 7 = very much like my partner

- Understanding
- Good job (or potential to achieve)
- Charismatic
- Supportive
- Financially secure (or potential to achieve)
- Outgoing
